# Determinants of minute ventilation-carbon dioxide production relationship in Chagas cardiomyopathy

**DOI:** 10.1590/0037-8682-0047-2021

**Published:** 2021-11-15

**Authors:** Lucas Frois Fernandes Oliveira, Janaina Martins Andrade, Pedro Henrique Scheidt Figueiredo, Matheus Ribeiro Ávila, Whesley Tanor Silva, Marcus Vinicius Accetta Vianna, Renato Guilherme Trede, Henrique Silveira Costa, Manoel Otávio Costa Rocha, Vanessa Pereira Lima

**Affiliations:** 1 Universidade Federal dos Vales do Jequitinhonha e Mucuri, Departamento de Fisioterapia, Diamantina, MG, Brasil.; 2 Universidade Federal dos Vales do Jequitinhonha e Mucuri, Programa de Pós-Graduação em Reabilitação e Desempenho Funcional, Diamantina, MG, Brasil.; 3 Universidade Federal dos Vales do Jequitinhonha e Mucuri, Faculdade de Medicina, Diamantina, MG, Brasil.; 4 Universidade Federal de Minas Gerais, Curso de Pós-graduação em Infectologia e Medicina Tropical, Belo Horizonte, MG, Brasil.

**Keywords:** Chagas cardiomyopathy, Chagas disease, Exercise test

## Abstract

**INTRODUCTION:**

The minute ventilation-carbon dioxide production relationship (VE/VCO_2_ slope) is among the main prognostic factors of Chagas cardiomyopathy whose determinants remain unknown.

**METHODS:**

Seventy-eight patients with Chagas cardiomyopathy were evaluated using clinical assessment, cardiopulmonary exercise test, echocardiography, and International Physical Activity Questionnaire.

**RESULTS:**

Age, functional class, International Physical Activity Questionnaire score, and dilated cardiomyopathy with systolic dysfunction were independent determinants of VE/VCO_2_ slope, and these variables explained 63% of its variance.

**CONCLUSIONS:**

The VE/VCO_2_ slope was related to age, functional class, physical activity level, and dilated cardiomyopathy with systolic dysfunction in patients with Chagas cardiomyopathy.

Chagas disease is defined as an acute or chronic infectious condition caused by the protozoan *Trypanosoma cruzi*. An estimated 6-7 million people are infected by the parasite worldwide, mostly in Latin America. Up to 30% of chronically infected people will develop cardiac abnormalities due to Chagas disease^1^. The cardiac form, Chagas cardiomyopathy (ChC), is the most common and severe clinical manifestation of the disease in which patients usually develop thromboembolism, malignant arrhythmias, and heart failure^2^. In ChC, even in the early stages of cardiopathy, intense fatigue, progressive dyspnea, and reduced functional capacity^3^ contribute to exercise intolerance.

The cardiopulmonary exercise test (CPET) is the gold standard for assessing functional capacity. In patients with heart failure, peak oxygen uptake (VO_2peak_) provides important clinical information. However, in recent decades, other CPET variables have demonstrated a strong prognostic value in these patients, such as the relationship between ventilation (VE) and carbon dioxide production (VCO_2_) expressed as the VE/VCO_2_ slope^4^. In a review, Arena, Myers, and Guazzi^5^ reported that an elevated VE/VCO_2_ slope was linked to ventilation-perfusion abnormalities. The authors also demonstrated that the VE/VCO_2_ slope and VO_2peak_ were the most well-established predictors of mortality in patients with heart failure.

In the setting of ChC, Ritt et al.^6^ reported that the VE/VCO_2_ slope was the only independent predictor of a worse prognosis (cut-off value of 32.5) among the variables evaluated by the CPET. However, unlike heart failure, few studies have evaluated the VE/VCO_2_ slope in patients with ChC^7^, and no study has demonstrated the determinants of the VE/VCO_2_ slope in this population. Thus, the present study aimed to verify the clinical, echocardiographic, and functional determinants of the VE/VCO_2_ slope in patients with ChC.

This cross-sectional study verified the determinants of VE/VCO_2_ slope in patients with ChC recruited from an outpatient reference center for Chagas disease. The study was approved by the Institutional Ethics Committee (approval number: CAAE 03993912.6.0000.5149) and performed in accordance with the Declaration of Helsinki. All patients provided written informed consent before participating.

Inclusion criteria included positive serology for *Trypanosoma cruzi* and the presence of arrhythmias and intraventricular and atrioventricular conduction disorders with or without left ventricular dysfunction (compatible with ChC). Patients with cardiopathy of any other cause, with respiratory, neurological, or systemic comorbidities that affect the results of exercise testing, or those who were unable to perform the CPET were excluded. 

All patients underwent clinical evaluation, echocardiography, physical activity level identification using the International Physical Activity Questionnaire (IPAQ), and CPET in the same week two days apart. The investigators were blinded to the experimental results.

All patients underwent a clinical evaluation by a cardiologist that included a structured anamnesis and physical examination protocol, with heart rate, blood pressure, and anthropometric data (weight, height, and body mass index). During the anamnesis, patients were also asked about their current medications and evaluated according to New York Heart Association (NYHA) functional class.

The CPET was performed on a treadmill using the metabolic analysis system MetaLyzer 3B (Cortex Medical, Leipzig, Germany). The VO_2peak_ and VE/VCO_2_ slope were obtained according to current guidelines^8^, and the highest values achieved during the test for both variables were considered in the analysis. An impaired VE/VCO_2_ slope was defined as values equal to or greater than 32.5^6^. The percentage of heart rate achieved during the test versus the maximal predicted value was also verified. The echocardiographic assessment was performed according to American Society of Echocardiography recommendations^9^. The target variables were the left ventricular ejection fraction (LVEF) and left ventricular end-diastolic diameter (LVDd). The LVEF was determined using Simpson’s rule. Dilated cardiomyopathy with systolic dysfunction was defined as a dilated left ventricle (LVDD value higher than 55 mm) with impaired ventricular systolic function (LVEF value less than 54% and 52% for women and men, respectively). The physical activity level was verified by the IPAQ, which estimates the time spent weekly in physical activity and classifies the patient’s physical level as sedentary (score 1), moderately active (score 2), or physically active (score 3)^10^.

The statistical analysis was performed using Statistical Package for the Social Sciences (SPSS) software, version 17.0. Data distribution was verified using the Kolmogorov-Smirnov test. Continuous variables were expressed as means and standard deviations or medians and interquartile ranges, and categorical variables were expressed as absolute numbers and percentages. The determinants of the VE/VCO_2_ slope were verified by univariate and multivariate linear regression analysis and were included in the multivariate analysis with a p value below 0.1 in the univariate model. To derive an equation to predict the VE/VCO_2_ slope in cases in which CPET findings were not available, the variables evaluated during the effort were not included in the univariate and multivariate models. The significance level was set at 5%.

A total of 78 patients were enrolled and evaluated in this study. The mean VE/VCO_2_ slope was 26.5±9.0. Twenty-one patients (30%) showed an impaired VE/VCO_2_ slope. The patients’ demographic, clinical, echocardiographic, and functional features are presented in Table 1.

**TABLE 1:** Characteristics of the sample (N=78).

Variable	Value	
	n	%
Age (years)*	52.8	10.1
Sex		
Male	35	44.9
Female	43	55.1
NYHA functional class		
I	44	56.4
II	25	32.1
III	9	11.5
BMI (kg/m^2^)^#^	26.6	22.7-29.3
HR (bpm)^#^	68.0	59.5-77.0
SBP (mmHg)^#^	106.0	100.0-130.0
DBP (mmHg)^#^	70.0	60.0-80.0
Physical activity level		
Sedentary	12	15.4
Insufficiently active	37	47.4
Moderately active	29	37.2
VO_2peak_ (mL.kg.min)*	23.1	6.8
VE/VCO_2_ slope*	26.5	9.0
% HR achieved*	82.8	13.4
LVEF (%) ^#^	58.0	40.5-64.0
LVDd (mm) ^#^	52.3	46.3-61.0
Systolic function		
Dilated cardiomyopathy with systolic dysfunction	28	35.9
Preserved systolic function	50	64.1

*Values shown as mean and standard deviation. #Values shown as median and interquartile range. **BMI:** body mass index; **DBP:** diastolic blood pressure; **HR:** heart rate; **LVDd:** left ventricular end-diastolic diameter; **LVEF:** left ventricular ejection fraction; **NYHA:** New York Heart Association; **SBP:** systolic blood pressure; VE/VCO_2_ slope, minute ventilation-carbon dioxide production relationship; VO_2peak_, peak oxygen uptake.

Table 2 shows that, in the univariate linear regression, age, sex, NYHA functional class, systolic and diastolic blood pressure, IPAQ score, and dilated cardiomyopathy with systolic dysfunction were associated with VE/VCO_2_ slope. In the final multivariate model, age, NYHA functional class, IPAQ score, and dilated cardiomyopathy with systolic dysfunction remained independent determinants of VE/VCO_2_ slope. Together, these variables explained 63% of the variance in the VE/VCO_2_ slope, which can be predicted by the equation VE/VCO_2_ slope = 47.69 + (0.41 × age) + (2.38 × NYHA) - (2.61 × IPAQ level) + (7.30 × dilated cardiomyopathy with systolic dysfunction; coded 1 for its presence and 0 for its absence).

**TABLE 2:** Univariate and multivariate analyses of the VE/VCO_2_ slope determinants in patients with ChC.

	Univariate model			Multivariate model*		
Variable	Beta coefficient	95% CI	P value	Beta coefficient	95% CI	P value
Constant	-	-	-	47.69	40.12 to 55.25	<0.001
Age	0.43	0.60 to 0.27	**<0.001**	0.41	0.28 to 0.54	<0.001
Male sex	6.88	3.17 to 10.52	**<0.001**	-	-	-
NYHA class	4.78	2.12 to 7.43	**0.001**	2.38	0.34 to 4.42	0.023
SBP	-0.32	-0.39 to -0.26	**<0.001**	-	-	-
DBP	-0.51	-0.67 to -0.34	**<0.001**	-	-	-
HR	-0.02	-0.14 to 0.10	0.758	-	-	-
BMI	-0.08	-0.54 to 0.37	0.713	-	-	-
Physical activity level	-5.77	-8.32 to -3.21	<0.001	-2.61	-4.59 to -0.64	0.010
Dilated cardiomyopathy with systolic dysfunction	10.06	6.47 to 13.65	<0.001	7.30	4.20 to 10.40	<0.001

Values highlighted in bold were included in the multivariate analysis (p < 0.1). *The adjusted r^2^ value for the model was 0.63. **BMI:** body mass index; **ChC:** Chagas cardiomyopathy; **CI:** confidence interval; **DBP:** diastolic blood pressure; **HR:** heart rate; **SBP:** systolic blood pressure.

To the best of our knowledge, this is the first study to verify the determinants of the VE/VCO_2_ slope in patients with ChC. The main finding of the present study was that dilated cardiomyopathy with systolic dysfunction, together with age, NYHA functional class, and physical activity level by IPAQ score were independent determinants of VE/VCO_2_ slope in patients with Chagas disease, explaining 63% of the variance. We also derived a model to predict the VE/VCO_2_ slope based on these parameters. The equation has potential value for estimating the VE/VCO_2_ slope when CPET findings are not available.

In the present study, the presence of dilated cardiomyopathy with systolic dysfunction explained 30% of the variation in the VE/VCO_2_ slope. We believe that the increase in the VE/VCO_2_ slope in the presence of dilated cardiomyopathy with systolic dysfunction is due to the muscle hypothesis. One of the main findings of dilated cardiomyopathy is the reduction of cardiac output and, consequently, decreased peripheral blood flow. This reduction in peripheral blood flow is associated with skeletal and respiratory myopathies, leading to increased ergoreflex and chemoreflex sensitivity^11^. The increased activity of the receptors induces ventilatory overactivation identified as the main factor responsible for the increase in the VE/VCO_2_ slope during exercise^12^. In addition, patients with cardiac chamber dilatation and chronic heart failure also presented with a restrictive lung pattern that could further compromise the ventilation and the VE/VCO_2_ slope^13^.

Age, functional class, and physical inactivity were also determinants of the VE/VCO_2_ slope. Aging is associated with the clinical worsening of heart disease as well as VO_2peak_ and VE/VCO_2_ slope deterioration in healthy individuals^14^. Functional class and physical inactivity were associated with reduced peripheral muscle mass. Impaired NYHA functional class and physical inactivity reduce capillary density, the proportion of type 1 fibers, and oxidative activity and increase the production of carbon dioxide during exercise. These muscle abnormalities can also lead to overactivation of the peripheral ergoreflex and an increased VE/VCO_2_ slope^15^.

The present study provided an equation to predict the VE/VCO_2_ slope based on its determinants. All variables were inexpensive and easy to determine except for the echocardiographic parameters. However, echocardiographic assessment is a part of the clinical routine of patients with ChC, and its features are often available. Because the VE/VCO_2_ slope is a strong prognostic marker, the model can be used to estimate this parameter in the risk stratification of patients with ChC when CPET results are not available. Finally, as a limitation, the present study predominantly included patients with a preserved VE/VCO_2_ slope; thus, further studies should be performed in patients with a severely impaired ventilatory efficiency. However, considering the setting of Chagas disease, the present study’s findings can provide valuable assistance with establishing the patient’s functional capacity.

In conclusion, ventilation efficiency assessed by VE/VCO_2_ slope was related to age, NYHA functional class, physical activity level, and dilated cardiomyopathy with systolic dysfunction in patients with ChC. These parameters have potential value for predicting the VE/VCO_2_ slope in these patients. To validate the model, a study using the proposed equation is desirable.
